# Variety of Cardiac Events in Hospitalized Chronic Kidney Disease Patients

**DOI:** 10.7759/cureus.18801

**Published:** 2021-10-15

**Authors:** Asfia Jabbar, Ruqaya Qureshi, Murtaza Dhrolia, Kiran Nasir, Aasim Ahmad

**Affiliations:** 1 Nephrology, The Kidney Centre Postgraduate Training Institute, Karachi, PAK; 2 Nephrology, The Kidney Center Post Graduate Training Institute, Karachi, PAK

**Keywords:** chronic kidney disease, supraventricular tachycardia, hyperkalemia, atrial fibrillation, cardiovascular disease, cardiovascular events

## Abstract

Objective

This study assessed the variety and frequency of various cardiovascular events in different stages of chronic kidney disease (CKD) patients who were hospitalized due to different causes.

Methods

This prospective cross-sectional observational analysis was conducted at the Department of Nephrology in The Kidney Centre Post Graduate Training Institute Karachi on all adult CKD (of all stages with or without dialysis) patients, who developed cardiovascular events during their hospital admission either in ward or ICU due to any cause between August 2020 and February 2021. Total of 765 patients got admitted in the given time period and among them, 290 patients developed various cardiovascular events. Baseline data, co-morbidities, clinical features, drug history and management were determined.

Results

There were a total of 290 patients in our study in which 154 (53.1%) were male and 136(46.9%) were female. Mean age was 57 ± 15.5. Our majority of patients were end-stage renal disease and on maintenance hemodialysis (n=119, 41%) while the most prevalent co-morbid condition was hypertension (n=227, 78.3%) followed by diabetes mellitus (n=204, 70.4%). The most frequent cardiovascular events in CKD patients was the atrial fibrillation 101(34.8%) while 37(12.8%) patients suffered ST-elevation myocardial infarction and supraventricular tachycardia. Patients who had high potassium levels (>5.2) most frequently suffered from atrial fibrillation (n=16, 28.1%) as compared to other cardiovascular events.

Conclusion

Patients with CKD are at increased risk of having several cardiovascular events. Numerous risk factors involved in the pathogenesis. Among the diverse causes, fluctuations in serum levels of various electrolytes are important causes as certain electrolytes disbalance can trigger various life-threatening cardiac arrhythmias.

## Introduction

Chronic kidney disease (CKD) is a worldwide public health problem; an estimated 200 million people have CKD. In the United States, African Americans have a four-fold additional risk of CKD compared to non-Hispanic white people and globally, people in the low-to-middle income countries like Asia and Sub-Saharan Africa have the maximum cases of CKD [1].

CKD has several stages, which are mainly based on measured or estimated glomerular filtration rate (GRF). In stage 1 there is kidney damage with normal or increased GFR (>90 mL/min/1.73 m²), in stage 2 there is a mild reduction in GFR (60-89 mL/min/1.73 m²), in stage 3a there is a moderate reduction in GFR (45-59 mL/min/1.73 m²), in stage 3b moderate reduction in GFR (30-44 mL/min/1.73 m²), in stage 4 there is a severe reduction in GFR (15-29 mL/min/1.73 m²) and in stage 5 GFR is less than 10ml/min/1.73m² in which patients needs renal replacement therapy either in the form of dialysis or renal transplantation.

There are various complications occur in CKD patients and these patients are at remarkably increased risk for both morbidity and transience from cardiovascular events (CVEs). It is familiar that various CVE rises as renal function decline [2] and when the patient reaches end-stage renal disease (ESRD), the prevalence of CVE further rises. Ohtake et al have reported that when patient started on dialysis approximately half of the patients with CKD have significant coronary artery stenosis regardless of patient having symptoms of cardiovascular disease (CVD) present or not and if patient is diabetic then percentage further rises to 90% [3].

Besides the co-morbidities like diabetes mellitus (DM), hypertension (HTN), dyslipidemia etc, it is well-known fact that CKD per se is a self-sufficient risk factor for CVD in fact individuals with CKD are most likely to die of CVD like then to develop ESRD [4].

There are various traditional and uremia-specific risk factors identified in patients with CKD. Traditional risk factors include HTN, smoking, DM, hyperlipidemia, Left Ventricular Hypertrophy (LVH) and male sex while albuminuria, anemia, hyperhomocysteinemia, abnormal metabolism of calcium and phosphorous, oxidative stress, fluid overload and malnutrition are well-known uremia related risk factors [4]. Novel threatening agents are endothelial dysfunction, Asymmetric Dimethyl Arginine (ADMA) and decreased nitric oxide. Besides risk factors, three pathological forms of CVD are highly common in patients with CKD. The first is LVH, the second is atherosclerosis and the third is arteriosclerosis of larger vessels.

Patients with CKD in either stage developed enormous cardiovascular incidents even when patient admitted for some other reason in the hospital, during their hospital stay these patients are at higher risk for developing certain cardiovascular complications. The spectrum of CVD in CKD patients includes ischemic heart disease (IHD), congestive cardiac failure (CCF), atrial and ventricular arrhythmias and peripheral vascular disease. Initially, it was thought that CVE limits to end-stage population but after study done by Go et al that adjusted heart rate for CV events increased inversely with the estimated GFR [2]. The USRDS 2014 annual report concluded that the prevalence of CVD is double in patients with CKD (69.8 vs 34.8%). Manjunath et al [5] quantified the risk of CVD and showed that for every 10ml/min decline in GFR the risk of CVE increased by 5%. As far as the presence of cardiac symptoms, the classical triad is usually not found in CKD patients and for diagnosing the CVE in these patients, ECG, cardiac biomarkers and echocardiography can be used as a diagnostic tool in these patients.

In many studies which were done on CKD patients who are not on dialysis, after correction of classical and nontraditional risk factors, there was still the great impact of CKD alone in causing CVD [6]. Study done by Go et al, in 2004 also concluded that there is documented linked between decrease GFR and CVE and therefore risk of hospitalization rises in these patients [2].

 CKD patients have a high risk of CVE either patient admitted for any reason as these patients have many risk factors so these patients at more prone to develop CVE so it is important to identify these patients and we should keep in mind the precipitating factors responsible for enhancing the CVEs.

## Materials and methods

A prospective cross-sectional observational analysis was conducted after getting approval from The Kidney Centre Post Graduate Training Institute Hospital Ethical Review Committee (ERC Reference number 93-NEPH-062020). We included all the adult CKD patients (of all stages with or without dialysis) who developed CVE during their admission either in ward or ICU due to any cause between August 2020 and February 2021, we excluded the patients who were admitted with acute kidney injury. The study included a total of 290 patients. The baseline data collected were age, sex, gender, etiology, stages of CKD, traditional risk factors like history of DM, HTN, dyslipidemia (defined according to ATP III guidelines or similar, if the patient received treatment with statins) [7] and smoking. Drug history was also taken like whether patients taking angiotensin-converting enzyme inhibitors (ACEI), ARBS (angiotensin receptor blockers), beta-blockers, antiplatelets and statins.

Clinical history was also observed which includes various clinical symptoms occurred during cardiac events like developed shortness of breath (SOB) and chest pain. Blood pressure and pulse were also observed during and after CVE. Cause of admission and which CVE occurred, also determined. Following laboratory parameters were also assessed before, during and after CVE: complete blood count (CBC), calcium, potassium phosphorus and magnesium. After the CVE, patient needs medical management or surgical intervention also determined.

The data was entered and analyzed on SPSS version 22. Cleaning and coding of data (data cleaning is the process of detecting and correcting these coding errors. There are two types of data cleaning that needs to be performed on data sets. They are possible code cleaning and contingency cleaning. Both are crucial to the data analysis process) was done prior to analysis. Categorical variables were expressed in frequency with percentage while mean± STD and median with IQR were computed for continuous variables. Association between variables was assessed by Chi square test. P-value of <0.05 was considered significant.

## Results

There were total of 290 patients in our study in which 154(53.1%) were male and 136(46.9%) were female. Mean age was 57 ± 15.5 with a minimum of 18 and a maximum of 90 years. Our majority of patients were ESRD and on maintenance Hemodialysis (HD) 119(41%) while the most prevalent comorbid was HTN 227(78.3%) (Table 1).

**Table 1:** Baseline parameters of chronic kidney disease patients. IQR: interquartile range; CKD: chronic kidney disease; DM: diabetes mellitus; HTN: hypertension; HD: hemodialysis.

	Mean ± SD	Median	IQR/n (%)
Age	57 ± 15.5	58.5	21
Weight	65.2 ± 10.4	67	57.8
Systolic BP before cardiac event	130.6 ± 21.5	132	21
Diastolic BP before cardiac event	79.4 ± 14.2	80	20
Systolic BP during cardiac event	127.3 ± 31	126	40
Diastolic BP during cardiac event	76.9 ± 18.6	80	25
Pulse before cardiac event	94.6 ± 13.2	98	13
Pulse during cardiac event	115.4 ± 34.6	127.5	50
		Number (n)	Percentage (%)
Gender	Male/Female	154/136	53.1/46.9
Smoker		81	27.9
Stage of CKD	II-III	20	6.9
	IV	59	20.3
	V	92	31.7
	V on HD	119	41
Duration	<1 year	37	12.8
	1-5 years	37	2.8
	>5 years	45	15.5
Frequency of HD/week	2/week	71	24.5
	3/week	48	16.6
Comorbid	DM	204	70.4
	HTN	227	78.3
	Dyslipidemia	156	53.8
Cause of CKD	DM	131	45.2
	Obstructive nephropathy	47	16.2
	Glomerulonephritis	22	7.6
	Unknown	90	31

Family history of cardiac disease was positive in 47(16.2%) patients and 100(34.5%) patients had a history of previous cardiac event. The most common cause of hospital admission was fluid overload in these patients 85(29.3%) followed by sepsis 72(24.8%) and urinary tract infection (UTI) (n=49, 16.9%). SOB was the most predominant symptoms of CVE in our patients 174(60%), on the other hand, 64(22.1%) patients suffered with chest pain during cardiac event. Low ejection fraction (EF) was found typically in echocardiography in these patients 109(37.6%). Majority of our patients recovered and discharged 190(65.5%), 49(16.9%) succumbed to death, while 51(17.6%) referred for any intervention and found alive on follow-up visits (Table 2).

**Table 2:** Clinical parameters of cardiac disease patients. SOB: shortness of breath; EF: ejection fraction; UTI: urinary tract infection; ACE: angiotensin-converting enzyme.

Clinical parameters of cardiac disease patients		n (%)
Positive F/H of cardiac event		49(16.2)
Positive H/O cardiac event		100(34.5)
Positive H/O cardiac intervention		38(13.1)
On ACE inhibitor		67(23.1)
On beta blocker		98(33.8)
On anti-platelet		164(56.8)
On statin		153(53.8)
Chest pain during cardiac event		64(22.1)
SOB during cardiac event		174(60)
Troponin leaked during cardiac event		76(26.2)
Echo cardiography findings	Low EF	109(37.6)
	Normal EF	101(34.8)
	Valvular lesion	75(25.9)
	Other	5(1.7)
Cause of hospitalization	Fluid overload	85(29.3)
	Acidosis/sepsis	72(24.8)
	UTI	49(16.9)
	Pneumonia	36(12.4)
	Aging problems	22(7.6)
	Dehydration	13(4.5)
	Obstructive nephropathy	7(2.4)
	GN	6(2.1)
Precipitating factors found		167(57.6)
Outcome	Recovered	190(65.5)
	Died	49(16.9)
	Referred for intervention	51(17.6)

Laboratory parameters of patients before and during CVE are shown in Table 3.

**Table 3:** Laboratory parameters of chronic kidney disease patients developed cardiovascular events.

Laboratory parameters	Mean ± SD	Median	IQR
Hb before cardiac event	8.6 ± 1.9	8.7	2.7
Hb during cardiac event	8.8 ± 1.6	8.7	2.2
TLC before cardiac event	12.7 ± 7.4	11.4	8.3
TLC during cardiac event	13.3 ± 7.7	12.2	8.5
Platelet before cardiac event	222.5 ± 90	222	101
Platelet during cardiac event	218 ± 92.7	222	110
K before cardiac event	4.5 ± 0.99	4.4	1.23
K during cardiac event	4.4 ± 1	4.3	1.4
Ca during cardiac event	8.2 ± 1.4	8	1.7
Mg during cardiac event	1.6 ± 0.36	1.5	0.47
PO4 during cardiac event	6.6 ± 2.5	6.6	3.3

**Figure 1:**
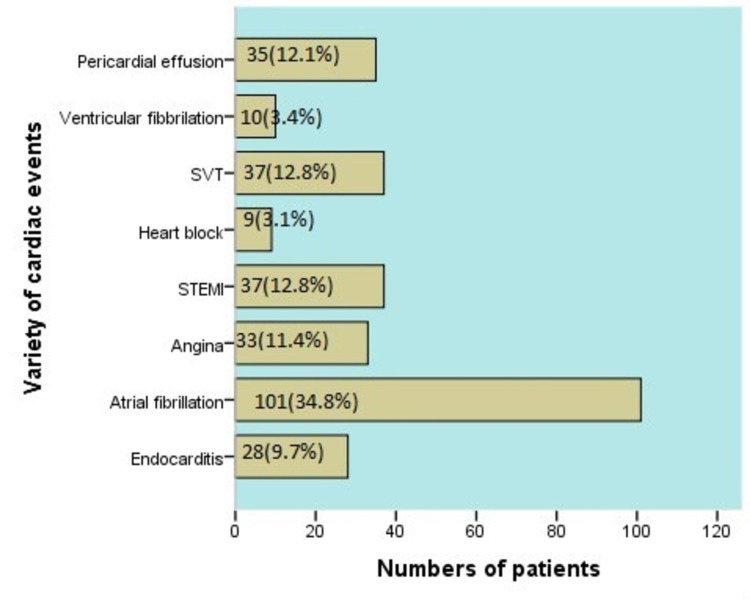
Variety of cardiac events.

The most prevalent cardiac event in CKD patients was the atrial fibrillation (AF) (n=101, 34.8%) while 37(12.8%) patients suffered from ST-segment myocardial infarction and supraventricular tachycardia (SVT) (Figure 1).

In our study patients who had increase potassium levels (>5.2) most frequently suffered from AF (n=16, 28.1%) as compared to other CVE.

**Figure 2:**
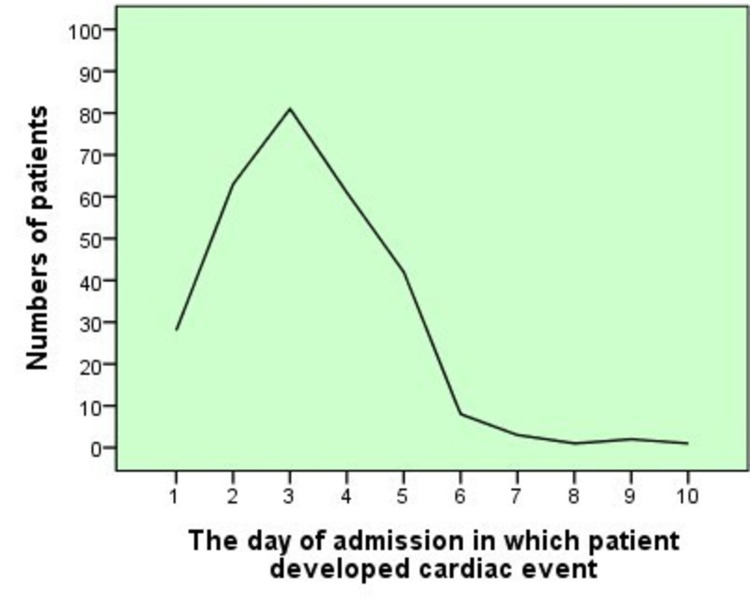
Day of admission for cardiac event.

Mainly the patients had encountered cardiac event on the third day of their admission 81(27.9%) on the other hand 63(21.2%) patient’s experienced cardiac illness on second day of hospitalization (Figure 2).

Type of CVE was highly significantly associated with the outcome of patients (p < 0.001). The majority of patients who suffered with endocarditis, recovered 14(50%) while 9 (32.1%) died, same as the recovery was predominantly high in patients who developed AF (n=92,91.1%), angina (n=25,75.8%), SVT (n=31,83.8%) and pericardial effusion (n=26,74.3%) respectively. On the other hand, all patients with Ventricular fibrillation (n=10,100%) and majority (n=5, 55.5%) of patients with heart block died (Table 4).

**Table 4:** Association of type of cardiac event with outcome.

Type of cardiac event	Outcome of patients				p-value
	Recovered	Died	Referred	Total	
Endocarditis	14(50)	9(32.1)	5(17.9)	28(9.7)	<0.001
Atrial fibrillation	92(91.1)	3(3)	6(5.6)	101(34.8)	
Angina	25(75.8)	1(3)	7(21.2)	33(11.4)	
STEMI	2(5.5)	11(29.7)	24(64.9)	37(12.8)	
Heart block	0	5(55.6)	4(44.4)	9(3.1)	
SVT	31(83.8)	6(16.2)	0	37(12.8)	
Ventricular fibrillation	0	10(100)	0	10(3.4)	
Pericarditis	26(74.3)	4(11.4)	5(14.3)	35(12.1)	

## Discussion

In patients with CKD, CVEs are twice as common in the general population and manifestated as coronary artery disease, heart failure, arrhythmias and heart block and it advances as GFR declines especially when GFR gets below 15 ml/min [2]. CVD is the major cause of mortality and morbidity in patients with CKD and especially who are on dialysis and they carry a substantial mortality and expected lifetime expectancy reduced by >50% and mortality accounts for 40 to 50% of all deaths in advanced stages of CKD compared with 26% in cohorts with normal kidney function [8]. We found that 119(41%) of the patients who developed different CVE were on HD and 92(31.7%) patients were of stage V (not on HD).

A meta-analysis of the relationship between CKD patients (non-dialysis dependent) and the risk factor for all cause and CV mortality involving 1371990 patients demonstrated that with declining GFR there is an augmented rise in absolute risk of death even after improvement in other risk factors [5], thus the CKD should be considered as one of the strongest predisposing factors for the development of CVD.

CKD is associated with traditional (age, sex, DM, BP and dyslipidemia) and non-traditional (GFR albuminuria, anemia ,phosphorus-calcium metabolism) cardiovascular (CV) risk factors [9]. DM and HTN are the most common causes of CKD but ESRD per se also responsible for CVD and CKD worsens the CV outcomes irrespective of other co-morbidities [10].

There are various pathogenesis involved in CVD with CKD. Chronic systemic inflammatory burden and oxidative stress have been connected to the pathogenesis of plaque formation and its subsequent rupture [11], both are associated with the worst CV outcomes. With decline in GFR, uremic toxins remained unclear and cause toxic effects on function of cells involved in myocardium and vessel function [12] and inhibition of leukocyte activity. CKD patients have a defective immune system which also triggers a micro inflammatory response and atherosclerosis. P cresol and indoxyl-sulphate are uremic toxins which gathered in CKD patients and exert a pro-inflammatory activity and activate an immune response and stimulates the progression of CKD and also lead to CVD [13].

Another pathogenic factor is metabolic acidosis, which is a common condition in ESRD patients which leads to malnutrition, bone disorders, CVE and even death [14]. Study done by Raikouand and Kyriaki [15] studied the association between low bicarbonate and CVD in patients on dialysis, they found that metabolic acidosis is significantly associated with diastolic dysfunction and increase pulse pressure.

In 1836, Richard Bright was the one who also reported the association of CKD with CVD [16] and in 1974, Linder et al [17] first time declared that patients on HD are predisposed to atheromatosis and they are more prone to develop CVE.

Hyperglycemia is strongly associated with the development of both CKD and CVD. In our study, DM is present in 204 (70.4%) patients who developed different CVE during their hospital stay. AF was the most common cardiac event (n=43 32.8%) occurred in patients who have DM for more than 10 years followed by angina (n=24 18.3%). Various studies done which showed a strong association of DM with various CVE [18-19]. Contrary to these studies, study done by Chio Okuyama et al in 2019 did not found a significant difference in CVE in patients with and without DM [20].

There are various studies regarding the effect of strict glucose control on CVE, in advance trial which was done on 11,000 patients with type II DM with rigorous strict control compared with standard or classic therapy leads to reduction in macro and microvascular events [21], while Accord trial which was done on 10000 patients with type 2 diabetics was not able to reveal any benefit of intensive sugar control on reduction of major CVE [22].

Besides DM, HTN is the most prevalent condition in CKD population also proved by our study as 78.3% of the patients have HTN as their co-morbid condition. Prolonged uncontrolled HTN leads to LVH and reduced EF. LVH occurs in CKD due to after load-related factors including arterial stiffness and systolic HTN which subsequently lead to LVH and reduced EF [23]. Second factor is expansion of intravascular volume in CKD patients which leads to volume overload and consequently causes left ventricular remodeling [23].

We also found low EF in 109(37.6%) patients. Valvular disease especially mitral and aortic valve disease has a strong effect on outcomes in patients with CKD [24]. In stage IV CKD, around 88%-99% of the patients have valvular calcification present in their echocardiography report [25]. We also found different valvular lesions in our patients in a frequency of 25.9%

After DM and HTN, dyslipidemia is the third most important risk factor for both CKD and CVD. High cholesterol levels are associated with several CVE, study done by Jose M Valdivev in 2017 showed an association of raised cholesterol with the occurrence of various CVE [26]. Various statins used in CKD and CV patients. In our study 153(52.8%) patients were using different statins. Atrovastatin is one of the statins which when used at a higher dose is associated with an increase in GFR and decrease in CVEs [27].

In our research most common cause of hospitalization was fluid overload (n=85, 29.3%), followed by acidosis (n=72, 24.8%), UTI (n= 49 ,16.9%) and pneumonia (n=36, 12.4%).

Besides anti-cholesterol therapy, study many patients (n=164, 56.6%) were already on different anti-platelets in our study group. In CKD with CVD a prognostic benefit of antiplatelets is unclear and it sometimes increases the risk of bleeding events possibly outweigh the potential benefits [28].

Beta-blockers were used by 98 (33.8%) patients. A meta-analysis of several interventional studies with beta-blockers in patients with CKD (stage III-IV) concluded that patients got benefitted from this therapy [29]. Another potential beneficial drug is ACEI which is used by 67(23.1%) patients in our study. In the Swedish cardiac insufficiency registry, 2,410 patients with CKD were examined, whose GFR was less than 30 ml/min with and without ACEI, conclusion revealed that in patients with ACEI, one-year mortality rate lessened as compared to patients who were not on ACEI [30].

Patients with CKD have specific electrolyte disturbances associated with CVE, i.e., high potassium can predict the occurrence of CVE and very high K predisposes to ventricular arrhythmias and sudden cardiac death [26]. In our study patients who had increase potassium levels (>5.2) most frequently suffered from AF (n=16, 28.1%) as compared to other cardiac events. Overall, we found AF as most common cardiac event occurred in our study (n=101, 34.8%) followed by SVT (n= 37, 12.8%) and angina (n=33, 11.4%). AF is a more common event that occurred in dialysis patients (n=39, 38.6%), while various studies showed the prevalence of AF is around 15-20% in dialysis population.

Increased phosphorus also associated with increased risk of CVE [26] and increase mortality in HD patients and it also causes increased risk of vascular calcification and enhances the risk of progression of atheroma plaque. In our study, 75.5%(219) of the patients have increase phosphorus levels who developed the various CVE.

A prospective study done in 2013 which concluded that advanced CKD patients are at great risk for all-cause CV mortality, certainly CVD accounts for more than 50% of deaths in patients with CKD [5], we also concluded from our study that CKD patients are prone to develop different CVEs.

## Conclusions

CKD creates a pro-inflammatory state which contributes to atherosclerosis and subsequently predisposes to certain CVE. CKD is a major risk factor for CVD and the leading cause of mortality and morbidity especially in patients on dialysis also consistent with our study as the majority of patients were of dialysis. Several risk factors are responsible for the causation of CVEs in the CKD population, these risk factors should be addressed early to avoid further subsequent complications.
